# No mutations of FecB and FecG^H^ in Iranian Lory sheep

**Published:** 2013

**Authors:** Zaynab Shafieiyan, Ghodratollah Mohammadi, Abbas Jolodarzadeh, Sara Amiri

**Affiliations:** *Department of Clinical Sciences, Faculty of Veterinary Medicine, Shahid Chamran University of Ahvaz, Ahvaz, Iran.*

**Keywords:** FecB, FecG^H^, Lory sheep breed, PCR, RFLP

## Abstract

The Booroola fecundity gene (FecB) and growth differentiation factor 9 (GDF9) gene belong to the transforming growth factor β (TGF-β) superfamily. The mutations of these genes have additive effects on the prolificacy in sheep. The aim of the present study was to determine the possible mutations of FecB and FecG^H^ genes in Lory sheep breed of the Lorestan province, Iran. Sixty blood samples were collected and DNA was extracted from whole fresh blood. For detection of FecB and FecG^H^ mutations, the PCR products were incubated with *AvaII* and *DdeI* restricted enzymes. Based on the results we did not find the FecB and FecG^H^ mutations in this sheep breed population, so these mutations cannot the cause of the high prolificacy of Lory sheep breed and more study are needed to determine the genetic or environmental causes of high prolificacy of this sheep breed.

## Introduction

Transforming growth factor β (TGF-β) superfamily is one of the widespread genes that have major effects on the reproduction.^1^ Booroola fecundity gene (FecB) and growth differentiation factor 9 (GDF9) belong to this superfamily. 

The FecB locus is situated in the region of ovine chromosome 6 corresponding to the human chromosome 4q22-23,^2^ and contains the bone morphogenetic protein receptor 1B (BMPR1B) gene. The A to G transition at nucleotide position 746 of the cDNA sequence induces a no synonymous substitution of glutamine with an arginine corresponding to position 249 of the mature protein.^3^^,^^4^ Based on segregation of the ovulation rate in Merino and Romney flocks, the genotypes in the ewes have been classified as homozygous no carrier (FecB+/FecB+) with ovulation rate of 2 or less, heterozygous carriers (FecBB/FecB+) with ovulation rate of 3-4 and homozygous carriers (FecBB/FecBB) with more than 5 ovulations per estrous cycle. This increased ovulation rate of FecBB carriers is associated with a precocious maturation of a large number of antral follicles that ovulate at a smaller size than non-carrier follicles.^5^

The ovine GDF9 gene maps to chromosome 5 and contains 2 exons. Eight single nucleotide polymorphisms (SNPs) have been identified so far in sheep GDF9, labeled G1-G8. The G8 mutation, also indicated as FecG^H^ (high fertility), causes increased ovulation rate in heterozygous ewes, while homozygous ewes are sterile.^6^^,^^7^

Carriers of FecB mutation are containing an *AvaII* restriction site (G/GACC) in the PCR products, whereas products from non-carriers lack this site. To detect the mutation, the 190 bp products were incubated with *AvaII* enzyme. Products containing the BMPRIB mutation were digested to yield a 160 bp fragment, whereas non-carriers products remained uncut at 190 bp.^8^

For detecting of the FecG^H^ mutation based on the method described by Hanrahan *et al*., the 139 bp PCR products are incubated with *DdeI* enzyme. The wild type products could be cleaved by *DdeI* (C/TTAG) but the mutation type with GDF9 remained uncleaved.^6^

Lory sheep is one of the Iranian breeds in the Lorestan province in central of Iran. The population of this sheep in Lorestan province is more than 3 million sheep. Average live weight of the lamb is about 3.7 kg and the adult ranges between 55 to 60 kg with mean litter size of 1.4. However, this breed is the best known in the region for their meat and wool production.

The aim of the present study was to determine the mutations of FecB and FecG^H^ genes in Lory sheep breeds of the Lorestan province.

## Materials and Methods

Sixty blood samples were collected into a 5 mL EDTA contained vacuum tube and transferred to laboratory for DNA extraction within 2 hr. Total DNA extractions were made using modified salting out method from whole fresh blood. ^9^

The specific primers were purchased from TAG Copenhagen (Copenhagen, Denmark). The restriction endonucleases (RE) and other reagents were purchased from CinnaGen (Tehran, Iran). Based on previous studies, the specific primers were designed.^6^^,^^10^ The sequences of primers and RE are described in Table 1.

**Table 1:** Primers and conditions used for PCR-RFLP

**Gene**	**Primer sequence ** **(5́→3́)**	**Annealing temperature (** **˚C)**	**Restriction enzyme**
**FecB**	CCAGAGGACAATAGCAAAGCAAA CAAGATGTTTTCATGCCTCATCAACAGGTC	60	*AvaII*
**FecG** ^H^	CTTTAGTCAGCTGAAGTGGGACAAC ATGGATGATGTTCTGCACCATGGTGTGAACCTGA	62	*DdeI*

The amplification of the loci was carried out in a total volume of 25 μL reaction.^11^ The reaction contained the following constituents: 100 ng of DNA used as a template, 1X PCR Buffer, 0.2 mM of each dNTP, 0.5 μM of each primer, 1 IU Taq polymerase and 1.5 mM of MgCl_2_. PCR amplifications were performed in a thermal cycler (Bioer, Tokyo, Japan) by an initial denaturation of 5 min at 95 ˚C, followed by 35 cycles of 30 sec of denaturation at 94 ˚C, 30 sec of annealing based on table 1, 30 sec of extension at 72 ˚C and a final extension of 10 min at 72 ˚C. The amplified products were electrophoresed in 2% agarose gel and the DNA bands were visualized by ethidium bromide staining technique.

For detection of FecB and FecG^H^ mutations, the PCR products were incubated with *AvaII* and *DdeI* overnight. For restriction fragment length polymorphism (RFLP) test; 10 µL of PCR product, 3µL 10X buffer, 1 IU RE and 15 µL double-distilled water mixed and incubated in water bath 37 ˚C overnight. After incubation, the digested products were analyzed by 3% agarose gel and ethidium bromide. 

## Results

Based on the results, the FecB and GDF9 genes were amplified in all samples. The electrophoresis of the PCR products showed the bands of 190 bp and 139 bp for FecB and GDF9 gene, respectively.

**Fig 1:**
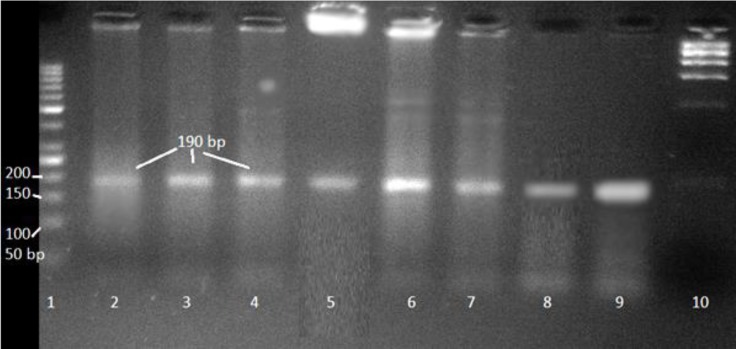
Agarose gel electrophoresis (2%) of allele specific BMPRIB PCR products digested by *AvaII* showing genotypes. Lane 1; 50 bp DNA marker, Lanes 2-9 represent different undigested products of samples from Lory sheep, lane 10 represents digested lambda DNA

**Fig. 2:**
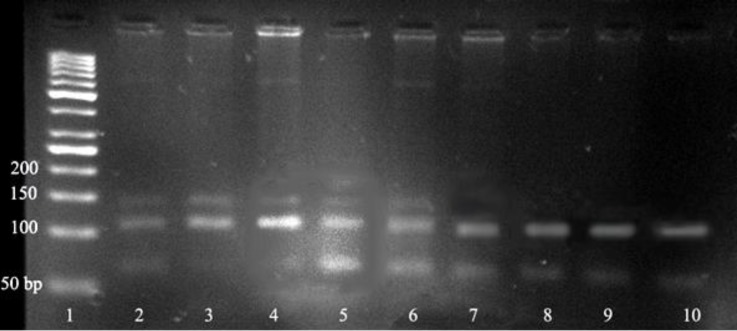
Agarose gel electrophoretogram for GDF9 mutation loci product digested by *DdeI* showing genotypes. Lane 1; 50 bp DNA marker. Lanes 2-10 represent different digested products of samples from Lory sheep

The electrophoresis of forced PCR-RFLP for detection of FecB and FecG^H^ have been shown in Figures 1 and 2, respectively. All of the 50 individuals were wild homozygote for FecB and FecG^H^ therefore none of the samples carried the mutation in FecB and FecG^H^ genes.

## Discussion

The FecX, FecB and FecG genes have been found to be closely associated with prolificacy in sheep.^1^^,^^12^^,^^13^ It has been reported that the litter size and ovulation rate in sheep increase with number of mutations in FecB gene.^1^ Ewes inheriting one copy of the Booroola gene from either of parents produced about 1.5 extra eggs and gave birth to about 1.0 extra lamb per lambing. Homozygous carriers produced about 3.0 extra eggs resulting in about 1.5 extra lambs per lambing.^1^ This increase in ovulation rate of FecBB carriers is associated with a precocious maturation of a large number of antral follicles that ovulate at a smaller size than non-carrier follicles.^5^

The FecB mutations have been reported in some of the world's most prolific sheep breeds as Australian Booroola Merino,^14^ Indian Garole,^10^ Indonesian Javanese,^10^ Small-tailed Han and Hu sheep of China.^15^ The FecG^H ^mutation was reported only in the Belclare and Cambridge sheep.^6^ In the present study, we utilized FecB and FecG^H^ as candidates, but did not find these mutations in the Lory sheep breed. 

The studies showed that maybe FecB and FecG^H^ were not the only ones responsible for the high prolificacy.^15^^,^^16^ Several investigations show that the FecBB allele is absent in low prolific sheep breeds,^17^ but it is also absent in many prolific sheep, such as Olkuska, Thoka and Woodlands breeds.^8^

Other studies showed that no mutations of FecB and FecG^H^ observed in some of the Iranian sheep breeds such as Lory-Bakhtiari, Arabic, Makui, Ghezel, Gharahgol, Iran Black, and Balochi. ^18^

It is recommended that other mutations of GDF9 and BMP15 genes need to be studied.^6^^,^^8^ For improvement of the Lory sheep and other Iranian flocks, introgression of big mutations such as FecB is recommended. Artificial insemination is one of the simple methods for introgression of the big mutations to the sheep flocks by importing sperm of mutation carriers such as Australian Booroola Merino, Indian Garole, Indonesian Javanese and Small-tailed Han Hu sheep of China.
